# Photodynamic therapy inhibit Fibroblast Growth Factor-10 induced keratinocyte differentiation and proliferation through ROS in Fibroblast Growth Factor Receptor-2b pathway

**DOI:** 10.1038/srep27402

**Published:** 2016-06-07

**Authors:** Maya Valeska Gozali, Fei Yi, Jia-an Zhang, Juan Liu, Hong-jin Wu, Yang Xu, Dan Luo, Bing-rong Zhou

**Affiliations:** 1 Department of Dermatology, the First Affiliated Hospital of Nanjing Medical University, Nanjing, 210029, China

## Abstract

5-aminolevulinic acid-photodynamic therapy (ALA-PDT) is known to be effective in several skin diseases such as acne, actinic keratoses, condyloma acuminata. However, some detailed mechanisms of ALA-PDT to treat these skin diseases still remain elusive. In this study, we aimed to investigate mechanism of ALA-PDT in *in-vitro* and *in-vivo* models. For *in vitro*, we use human keratinocyte cell line (HaCaT) cells. CCK-8 was used to detect cell proliferation activity, immunofluorescence and western blotting method to detect the content of keratin (K)1, K6, K16, protein kinase C (PKC), fibroblast growth factor receptor-2b (FGFR2b) protein, ELISA and RT-PCR to detect expression of interleukin (IL) 1α in the cell supernatant, and detect reactive oxygen species (ROS). For *in vivo*, we use 20 rabbits to induce hyperkeratosis acne model in their ear. Dermatoscope was used to see follicle hyperkeratosis and skin biopsy to analyze histology and immunohistochemical of PKC, FGFR2b, K1, K6 and K16. Results from this study suggest that ROS stimulated by ALA-PDT lead to inhibition of FGFR2b pathway in PKC downstream to cause reduction of IL1α expression, and eventually, keratinocytes differentiation and proliferation. Our data thus reveal a treatment mechanism of ALA-PDT underlying hyperkeratosis related dermatoses.

Clinical studies have reported over years that 5-aminolevulinic acid-photodynamic therapy (ALA-PDT) has effects in treatment of several skin keratosis such as acne, actinic keratosis, and human papiloma virus infectious skin diseases^1^,^2^,^3^,^4^,^5^,^6^. Abnormal differentiation of follicular keratinocytes with increased cornification is one of major roles in the pathogenesis of acne^7^. Some researchers have shown effectiveness of ALA-PDT for non-inflammatory acne lesion besides inflammatory lesion^4^,^8^. The follicular obstruction in non-inflammatory acne lesions was believed to be reduced by ALA-PDT through changing keratinocyte shedding and hyperkeratosis^2^. Recently, ALA-PDT was also shown successfully treated abnormal keratinization related dermatoses such as psoriasis and porokeratosis^9^,^10^,^11^. These clinical results suggest ALA-PDT may regulate in abnormal keratinocyte differentiation and proliferation. However, the underlying mechanisms were still unclear.

ALA is a precursor of protoporphyrin IX (PpIX), which is well known as a strong photo-sensitizer, and interconvert to PpIX via the heme biosynthetic pathway. When PpIX is activated by light of appropriate wavelength during PDT, reactive oxygen species (ROS) induces cell necrosis and apoptosis^12^,^13^ which offer a unique way of improving acne by selectively damaging the pilosebaceous unit and killing P. acnes^4^.

Fibroblast growth factor-10 (known as FGF-10 or KGF-2), another member of the FGFs family, is highly similar to keratinocyte growth factor in amino acidic sequence and tissue expression. FGF-10 promotes not only keratinocyte cell growth but also the differentiation program^14^. FGF-10 acted by binding with high affinity to the tyrosine kinase keratinocyte growth factor receptor (KGFR/FGFR2b)which is a splicing variant of the fibroblast growth factor receptor-2 (FGFR2)^15^. Increased FGFR2b-signaling is associated with acne in Apert syndrome and has been suspected to be of pathophysiological importance in acne vulgaris^16^. The physiologic role of the FGFR2b gets further support by the concept that several anti-acne agents may exert their therapeutic effects by downregulation of increased FGFR2b signal transduction such as benzoyl peroxide (BPO), tetracycline, erythromycin, and retinoids^17^. Previous study demonstrated ROS induce ligand-independent FGFR2b internalization and lysosomal degradation, thereby terminating FGFR2b signaling^14^. Besides that, Melnik *et al*. concluded that anti-acne mechanism of BPO is through ROS generation to suppress FGFR2b expression^17^, which further suggest that FGFR2b receptors in keratinocytes are likely to be impacted by oxidative damages.We thus hypothesized that ALA-PDT may target at FGFR2b pathway for treatment of acne. In this study, we found that ALA-PDT is involved in the inhibition of differentiation and proliferation of keratinocytes induced by FGF-10 through increasing ROS in FGFR2b pathway.

## Results

### No apoptotic effect of ALA-PDT in HaCaT cells

To observe whether ALA-PDT has any apoptotic effect in HaCaT cells, we do flow cytometry to quantify the apoptotic percentage in two groups: control group and ALA-PDT group. As shown in supplementary Figure, apoptotic percentage was quantified and compared to control (2,29%), ALA-PDT did not have any apoptotic effect to the cells (3,67%), the differences were not significant (P > 0.05) (Supplementary Figure a,b).

### Impact of ALA-PDT in FGF-10 induced differentiation and proliferation of HaCaT cells

**Figure 1:**
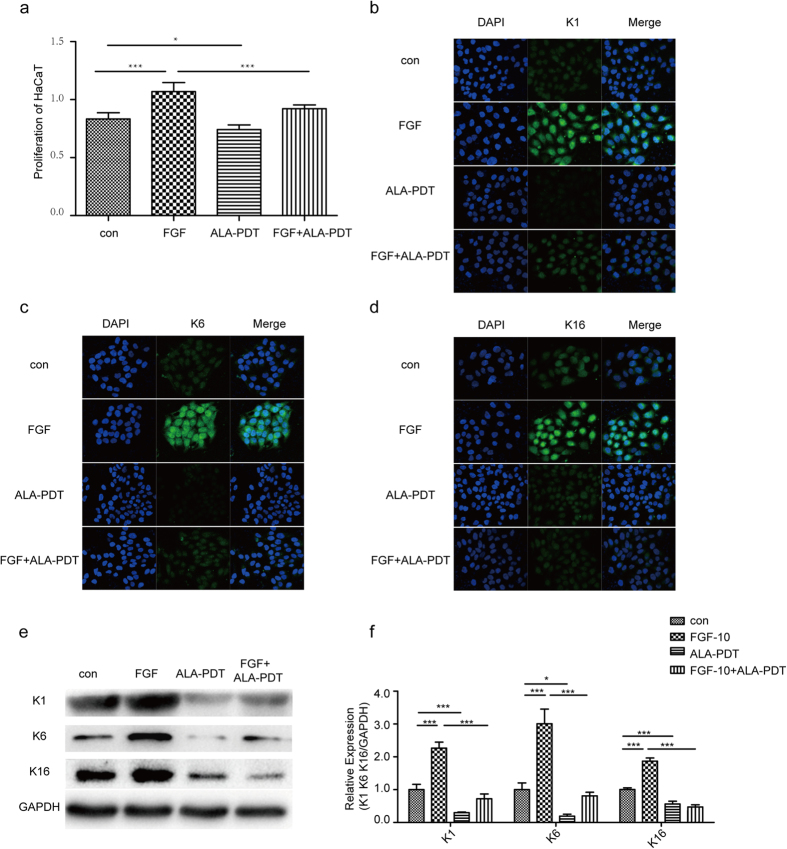
5-aminolevulinic acid-photodynamic therapy (ALA-PDT) inhibits FGF-10-induced proliferation and differentiation of HaCaT. Cells were treated with none as control, FGF-10, ALA-PDT, FGF-10 + ALA-PDT (FGF-10 condition: 100ng/ml; ALA-PDT condition: 1mM ALA and 3 J/cm^2^) and cell proliferation was analyzed by CCK-8 (**a**). ALA-PDT suppress keratinocyte differentiation and proliferation shown by immunofluorescence staining (**b–d**) and western blot analysis (**e,f**) of K1 as differentiation marker, K6 and K16 as proliferation markers (green). DAPI (blue) was used as a nuclear stain. Cropped blots are used and the blots have been run under the same experimental conditions. Protein expressions were quantified by used GAPDH as a control. The data represent the average of the three independent experiments. Values of *P < 0.05 and ***P < 0.001 were considered significant (mean ± SD). Bar = 100 μm, N = 3.

To investigate the effect of ALA-PDT on proliferation of HaCaT, we do CCK-8 assay. As shown in Fig. 1a, ALA-PDT decrease the proliferation of HaCaT as compared with control and FGF-10, P < 0.05. Western blot and immunofluorescence experiment were performed to identify the expression of K1 as differentiation marker, K6 and K16 as proliferation markers in HaCaT cell. As shown in Fig. 1b–d, immunofluorescence staining show the intensity of K1, K6, and K16 in FGF-10 group was significantly higher than that in control group. However, it was lower in ALA-PDT group than that in control and FGF-10 + ALA-PDT groups, P < 0.05. As shown in Fig. 1e,f, western blot results show K1, K6 and K16 proteins in FGF-10 group were increased significantly compared with the control group. Nevertheless, the increase in the expression of K1, K6, and K16 were abrogated upon ALA-PDT, P < 0.05.

### Impact of ALA-PDT in PKC downstream of FGFR2b signaling pathway

**Figure 2:**
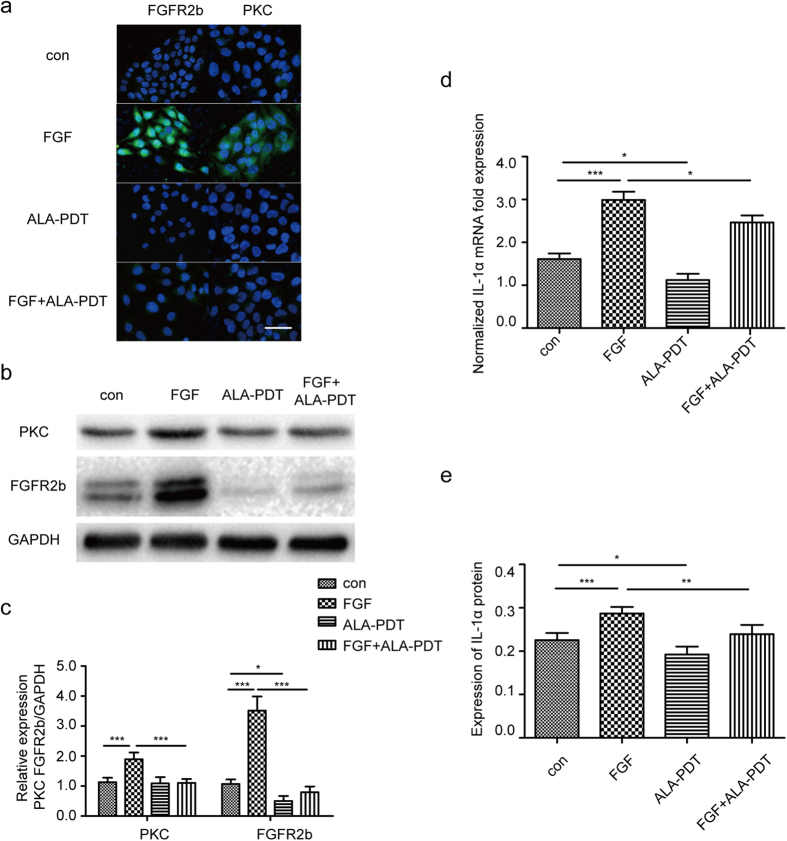
ALA-PDT suppressed FGF-10 induced IL1α expression, PKC and FGFR2b protein in HaCaT. Cells were treated with none as control, ALA-PDT, FGF-10, FGF-10 + ALA-PDT (FGF-10 condition: 100 ng/ml; ALA-PDT condition: 1 mM ALA and 3 J/cm^2^). Immunofluorescence staining (**a**) and western blot analysis (**b,c**) of FGFR2b and PKC shown that FGFR2b and PKC were suppressed by ALA-PDT. 4′,6-Diamidino-2-phenylindole (DAPI) (blue) was used as a nuclear stain. Cropped blots are used and the blots have been run under the same experimental conditions. Protein expressions were quantified by used GAPDH as a control. Expression levels of IL1α suppressed by ALA-PDT. mRNA of IL1α level was analyzed by RT-PCR (**d**) and the protein level was analyzed by ELISA (**e**). The data represent the average of the three independent experiments. Values of *P < 0.05, **P < 0.01, and ***P < 0.001 were considered significant (mean ± SD). Bar = 100 μm, N = 3.

To explore the underlying mechanism of ALA-PDT regulation, we demonstrate the expression of PKC and FGFR2b protein by Western blot analysis and immunofluorescence staining. Immunofluorescence assay of PKC and FGFR2b in FGF-10 group showed higher immunofluorescence intensity than that in control group. Moreover, it was lower in ALA-PDT group than that in FGF-10 + ALA-PDT groups, P < 0.05 (Fig. 2a). As shown in Fig. 2b,c, PKC and FGFR2b protein in FGF-10 group was increase significantly when compared with control and it was decrease significantly after ALA-PDT was added, P < 0.05. IL1α assessed by RT-PCR and ELISA showed that FGF-10 group increased significantly compare to control group, however, expression of IL1α was decreased after ALA-PDT, P < 0.05 (Fig. 2d,e).

### Effect of ALA-PDT in acne rabbit’s ear model

**Figure 3:**
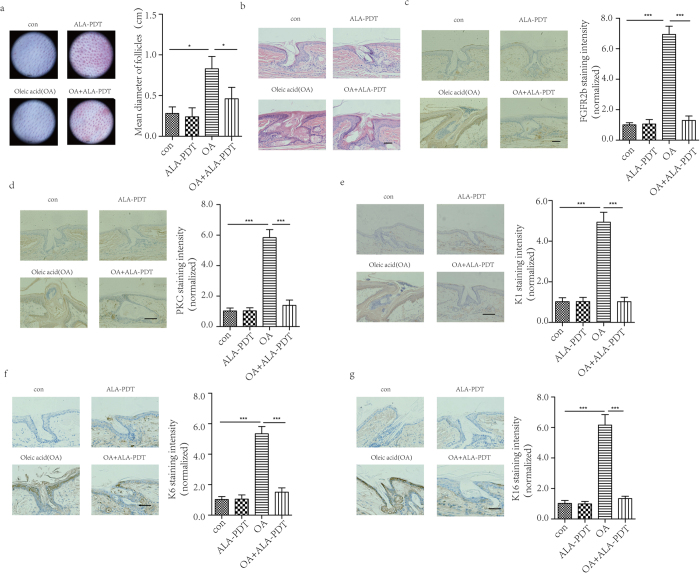
ALA-PDT decrease follicles size and expression of FGFR2b, PKC, K1, K6, K16 *in vivo*. Rabbit’s ear was treated with none as control, ALA-PDT twice in 14 days, topical oleic acid (OA) to induce acne model for 14 days, OA + ALA-PDT. After ALA-PDT, obstruction in pilosebaceous duct was reduced clinically (**a**) and histologically. Skin biopsy specimens were examined with hematoxylin-eosin (**b**) (original magnification ×200) and immunohistochemical staining demonstrated ALA-PDT reduced the accumulation of FGFR2b (**c**), PKC (**d**), K1(**e**), K6 (**f**), K16 (**g**) (Original magnification ×200). The data represent the average of the three independent experiments. Bar = 100 μm, N = 3. Values of *P < 0.05 and ***P < 0.001 were considered significant (mean ± SD).

An acne rabbit’s ear model was induced using OA topical application. In the OA group, enlarged hair pores were observed in the rabbit’s ear after OA application and were significantly higher than control group, P < 0.05. The hair pores were significant decrease in OA + ALA-PDT group, P < 0.05 (Fig. 3a). Histological examination of the hair pores showed markedly dilated hair follicles and hypertrophy of the sebaceous glands in the OA group, and these findings are characteristic of comedone formation. ALA-PDT significantly inhibited these histological changes as shown in Fig. 3b. Immunohistochemical analysis revealed staining intensity of FGFR2b in OA group was significantly higher than that in control group, P < 0.05. In OA + ALA-PDT group, it was significantly lower than in OA group, P < 0.05 (Fig. 3c). Moreover, PKC staining intensity in OA group showed higher intensity than that in control group and showed lower intensity after ALA-PDT, P < 0.05 (Fig. 3d). In addition, K1, K6, and K16 staining intensity were significantly higher in OA group than that in control group and significantly lower after ALA-PDT, P < 0.05 (Fig. 3e–g).

### Influence of PKC inhibitor (PKCi) and activator (PKCa) in FGF-10 induced differentiation and proliferation of HaCaT cells in PKC pathway

**Figure 4:**
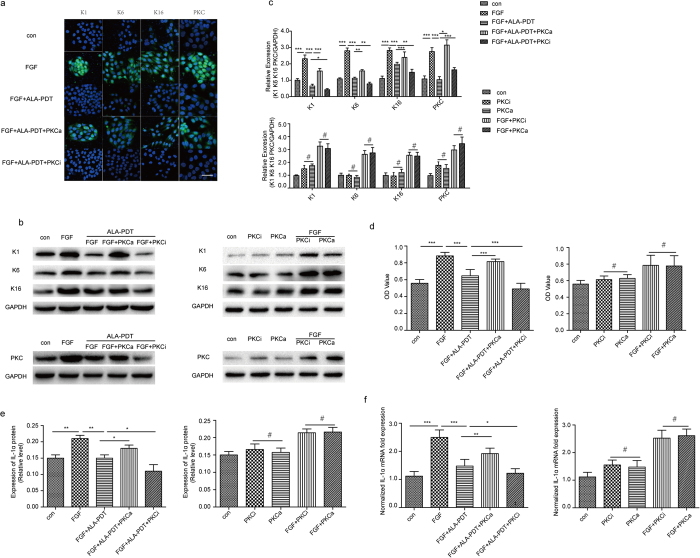
IL1α, PKC, differentiation and proliferation markers after ALA-PDT were downregulated/upregulated due to PKC inhibitor/activator. Cells were treated with none as control, FGF-10, FGF-10 + ALA-PDT, FGF-10 + ALA-PDT + PKC activator (PKCa), FGF-10 + ALA-PDT + PKC inhibitor (PKCi), con + PKC-i, con + PKC-a, FGF + PKC-i, FGF + PKC-a. (FGF-10 condition: 100 ng/ml; ALA-PDT condition: 1 mM ALA and 3 J/cm^2^, PKCa: 1 μmol/l and PKCi: 200 nmol/l for 1 hour before adding FGF-10). Immunofluorescence staining of K1, K6, and K16 (green) (**a**) were taken by confocal microscope and protein level was analyzed by western blotting (**b,c**) DAPI (blue) was used as a nuclear stain. Cropped blots are used and the blots have been run under the same experimental conditions. Protein expressions were quantified by used GAPDH as a control. Cell proliferation analyzed by CCK-8 (**d**). The protein level of IL1α was analyzed by ELISA (**e**) and mRNA was analyzed by RT-PCR (**f**). The data represent the average of the three independent experiments.Values of *P < 0.05, **P < 0.01, and ***P < 0.001 were considered significant (mean ± SD). Values of ^#^P > 0.05 were considered not significant (mean ± SD). Bar = 100 μm, N = 3.

To further prove ALA-PDT has impact in PKC pathway, we next examined the effect of the Staurosporine as PKC inhibitor and 12-O-tetradecanoyl-phorbol-13-acetate (TPA) as PKC activator in FGF-10 induced differentiation and proliferation of HaCaT after ALA-PDT was added. Western blot showed expression of K1, K6, K16 proteins in FGF-10 + ALA-PDT group significantly lower than that in FGF-10 + ALA-PDT + PKCa group and significantly higher than that in FGF-10 + ALA-PDT + PKCi group, P < 0.05 (Fig. 4b,c). Intensity of K1, K6, K16 expressions were visualized using confocal microscope (Fig. 4a). Furthermore, Staurosporine was worked synergistically with ALA-PDT to inhibit the proliferation of HaCaT and TPA significantly induced the proliferation, P < 0.05 (Fig. 4d). RT-PCR and ELISAindicated that IL1α expression was induced predominantly by PKCa, whereas PKCi effect in a decrease of IL1α, P < 0.05 (Fig. 4e,f).

### Effect of ROS in FGF-10 induced differentiation and proliferation of HaCaT cells

**Figure 5:**
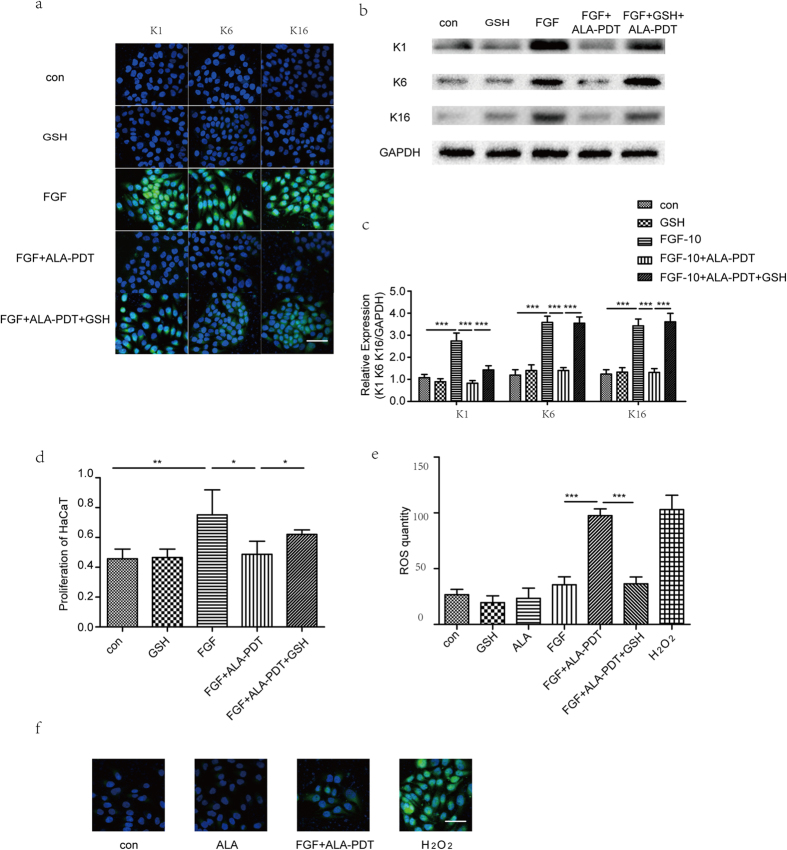
Intracellular reactive oxygen species (ROS) by ALA-PDT contributes in FGF-10 induced HaCaT differentiation and proliferation. Inhibition of ROS generation by antioxidant gluthatione (GSH) abolished inhibition of ALA-PDT in FGF-10 induced keratinocyte differentiation and proliferation. Immunofluorescence staining (**a**) and western blot analysis (**b,c**) of K1, K6, and K16 (green) were performed in 5 groups: control, GSH, FGF-10, FGF-10 + ALA-PDT, and FGF-10 + ALA-PDT + GSH (FGF-10 condition: 100 ng/ml; ALA-PDT condition: 1 mM ALA and 3 J/cm^2^, GSH: 5 mg/ml for 1 hour before ALA-PDT treatment). DAPI (blue) was used as a nuclear stain. Cropped blots are used and the blots have been run under the same experimental conditions. Protein expressions were quantified by used GAPDH as a control. Cell proliferation analyzed by CCK-8 (**d**). ALA-PDT induced intracellular ROS generation and ROS was quantified by flow cytometry and confocal microscopy (**e,f**). As a control, cells were treated with H_2_O_2_ (0.5 mM) for 30 minutes and ALA alone also evaluated for ROS generation. The data represent the average of the three independent experiments. Values of *P < 0.05, **P < 0.01, and ***P < 0.001 were considered significant (mean ± SD). Bar = 100 μm, N = 3.

Since our data indicate that ALA-PDT inhibit FGF-10 induced differentiation and proliferation of HaCaT, it is possible that ALA-PDT generated ROS inhibit FGFR2b activation. We measured intercellular ROS generation by FACS using DCFH-DA staining. As shown in Fig. 5e, HaCaT cells treated with ALA-PDT showed increased intracellular ROS production while ALA alone had little effect on fluorescent intensity. ALA-PDT-induced ROS generation was also visualized with confocal microscopy (Fig. 5e,f). In order to examine the potential involvement of ROS in the differentiation and proliferation response to ALA-PDT, we investigated that the effect of antioxidants, GSH on HaCaT differentiation and proliferation following ALA-PDT. Immunofluorescence staining of K1, K6, K16 showed higher intensity after add GSH 1 hour before ALA-PDT, P < 0.05 (Fig. 5a). Western blot analysis of K1, K6, and K16 protein in FGF-10 + ALA-PDT + GSH group were higher than that in FGF-10 + ALA-PDT group, P < 0.05 (Fig. 5b,c). Moreover, the proliferation of HaCaT in ALA-PDT + GSH were higher than in ALA-PDT, P < 0.05 (Fig. 5d), which shown GSH induced proliferation after ALA-PDT indicating that ALA-PDT induced ROS generation contributes to the regulation.

### Effect of ROS in PKC downstream of FGFR2b signaling pathway

**Figure 6:**
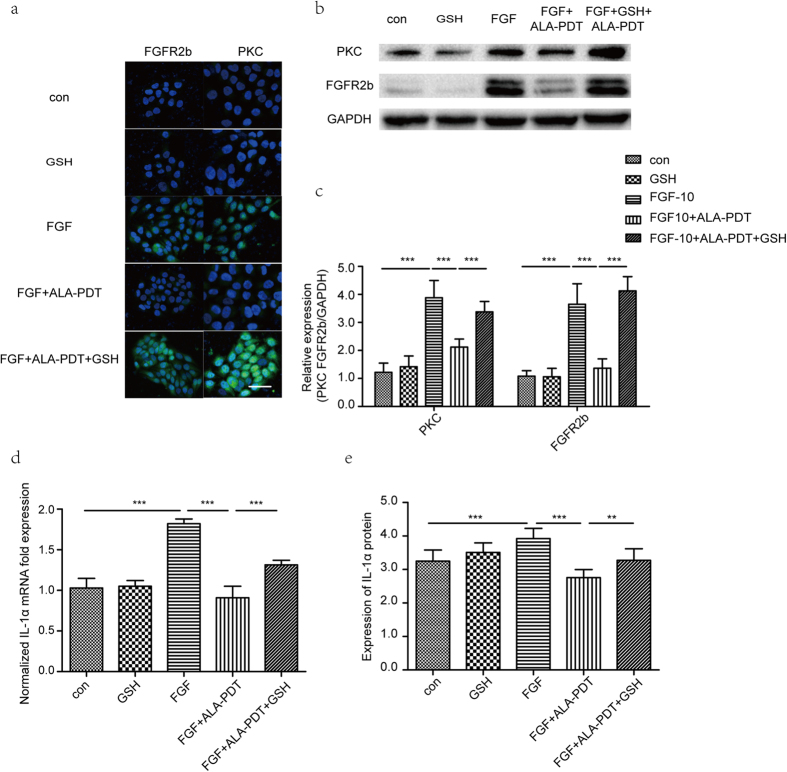
Intracellular ROS by ALA-PDT contributes in FGF-10 induced IL1αexpression, PKC and FGFR2b protein. Inhibition of ROS generation by antioxidant GSH abolished inhibition of ALA-PDT in FGF-10 induced IL1α expression, PKC and FGFR2b protein. Immunofluorescence staining (**a**) and western blot analysis (**b,c**) of FGFR2b and PKC were performed in 5 groups: control, GSH, FGF-10, FGF-10 + ALA-PDT, and FGF-10 + ALA-PDT + GSH (FGF-10 condition: 100 ng/ml; ALA-PDT condition: 1 mM ALA and 3 J/cm^2^, GSH: 5 mg/ml for 1 hour before ALA-PDT treatment). DAPI (blue) was used as a nuclear stain. Cropped blots are used and the blots have been run under the same experimental conditions. Protein expressions were quantified by used GAPDH as a control. mRNA of IL1α level was analyzed by RT-PCR (**d**) and the protein level was analyzed by ELISA (**e**). The data represent the average of the three independent experiments.Values of **P < 0.01 and ***P < 0.001 were considered significant (mean ± SD). Bar = 100 μm, N = 3.

Furthermore, we investigated whether ALA-PDT induced ROS generation has impact in FGFR2b and PKC pathway. As shown in Fig. 6a, ALA-PDT inhibition efficiently inhibited PKC and FGFR2b while significant increase occur when adding GSH, P < 0.05. Similarly, PKC and FGFR2b protein decrease considerably after ALA-PDT while GSH induced both proteins increased significantly, P < 0.05 (Fig. 6b,c). In addition, GSH suppressed ALA-PDT inhibition of IL1α expression, P < 0.05 (Fig. 6d,e).

## Discussion

In this study, we found that ALA-PDT can inhibit FGF-10 induced proliferation and differentiation of HaCaT cells and it generates intracellular ROS to suppress IL1α in FGFR2b pathway. We used HaCaT cells because it is spontaneously immortalized from a primary culture of keratinocytes, well characterized, often used as a suitable model system to study the *in vitro* events of keratinocyte proliferation and differentiation, also it used in research related to acne^18^,^19^,^20^,^21^.

Keratins are important marker for evaluating the stage of keratinocyte that have two alternative pathways open to them: differentiation that characteristically express K1 and proliferation express K6 and K16^22^,^23^,^24^. Hyperproliferation and abnormal differentiation of the follicular keratinocytes are believed to be the major contributor to early stages of acne pathogenesis^25^. The present novel *in vitro* findings of regulation effect of ALA-PDT on differentiation and proliferation of keratinocytes suggest its new role it in acne treatment. To address the issue if the *in vitro* finding also applies to the *in vivo* situation, an acne rabbit’s ear model was utilized. Confirming the *in vitro* results, ALA-PDT showed significant effects of reducing K1, K6, and K16 that reflecting similar efficacy *in vitro*, as well confirming the previous study on histology of human skin samples which concluded that one of the mechanisms of ALA-PDT in acne treatment was reducing follicular obstruction by changing keratinocyte shedding and hyperkeratosis^2^. Many acne treatments act through regulation of hyperproliferation and abnormal differentiation on follicular keratinocytes such as topical administration of retinoid reduce the expression of keratinocyte differentiation markers also keratin 6^26^ and a statisfactory clinical response to long term oral tetracycline treatment associated with the decreasing amount of keratin in the pilosebaceous duct^27^. We further confirm ALA-PDT may therefore have several modes of action that not only selectively damaging the pilosebaceous unit and killing *P. acnes*, it also has keratin regulation effect. Besides that, Smits *et al*. found that psoriatic lesions showed some evident clinical improvement during the ALA-PDT treatment period which was reflected in a statistically significant decrease in the clinical plaque severity score^11^. Several cases of classic porokeratosis such as porokeratosis of Mibelli treated with PDT successfully revealed^9^,^10^. Above clinical results indicate ALA-PDT regulates keratinization, however still need specific models to confirm it.

Elevated levels of pro-inflammatory IL1α have been detected in most open comedones of acne vulgaris^28^,^29^. External treatment with the IL1α protein leads to hyperproliferation and abnormal differentiation in isolated pilosebaceous units *in vitro*^30^,^31^. IL1α acts on infudibular keratinocytes to promote cornification and freshly isolated infudibula were found to express keratins 1, 6, and 16^32^. Reducing the expression of IL1α is the mechanism of all-trans-retinoic acid as one of the most effective agents preventing comedone formation and exhibiting comedolytic activity^17^. Our studies showing ALA-PDT suppresses the elevated IL1α production induced by FGF-10 which in accordance with its regulation on keratinocytes differentiation and proliferation. IL-1α has been reported to be induced by FGFR2b-signaling and its downstream^23^. The three major downstream FGFR2b-signaling cascades: the MAPK pathway-inducing cell proliferation and MMP expression, the PI3K/Akt- and Shh/MC5R pathway-inducing lipogenesis and terminal sebocyte differentiation, and the phospholipase Cg/PKC pathway-inducing IL-1α, and inflammatory reactions^33^. The previous study showed inhibition of the PKC pathway decreased the expression of IL1α gene which suggesting that the PKC pathway is upstream of IL1α gene expression^34^. In this present study, we found same effect with above findings that the IL1α was reduced when add PKC inhibitor. Our results suggest that ALA-PDT inhibit keratinocyte differentiation and proliferation through PKC pathway.

**Figure 7:**
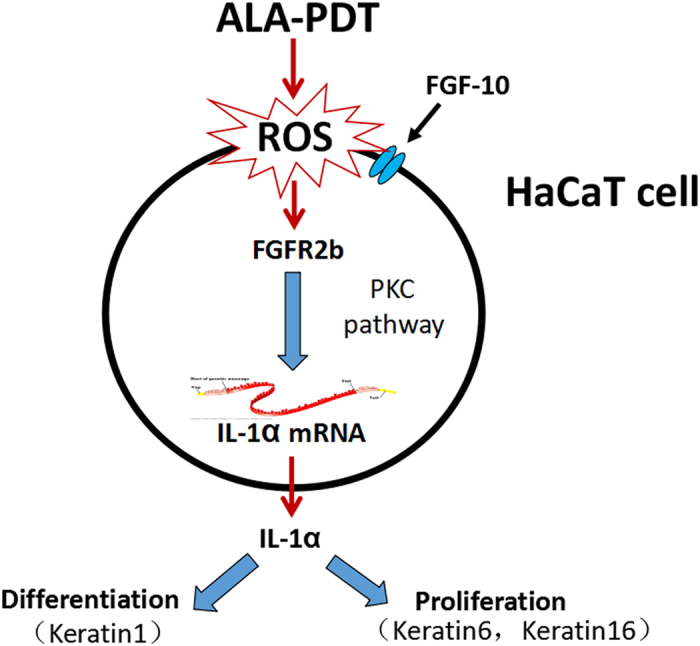
Synopsis of FGF-FGFR2b-mediated HaCaT signaling. FGF-10 stimulated HaCaT to activate FGFR2b signaling which increases IL1α expression through PKC-dependent pathway. HaCaT-derived IL1α induces expressions of K1, K6 and K16 thereby upregulating differentiation and proliferation of HaCaT. ALA-PDT generates ROS which were reversed the FGF-FGFR2b-mediated HaCaT signaling.

ROS has a major role in mechanism of ALA-PDT. It can cause decreased cellular proliferation and increased cytotoxicity such as in treatment of condyloma acuminata and several skin cancers^35^,^36^. However, low concentrations of ROS after PDT may activate cellular functions of dermal fibroblasts that lead to proliferation and activation of dermal fibroblast for the skin rejuvenation effects^37^. In our study, low concentration of ALA induce ROS production did not enough to cause cytotoxicity. We use western and immunofluorescence analysis to prove that ROS lead to significant reduction of FGFR2b. ROS production capable to impact FGFR2b such as a previous study reported that BPO is an effective oxidative agent for treating acne by inducing the formation of intracellular ROS which might downregulate increased FGFR2b signaling by lysosomal receptor degradation. Besides that, by addition of antioxidant such GSH to inhibit ROS, our results showed it can inhibit the capability ALA-PDT of suppressing FGFR2b. We suggest mechanism treatment of ALA-PDT is to produce low concentration of ROS which not lead to cytotoxicity while through suppressing FGFR2b signaling pathway to cause reduction of IL1α expression induce keratinocyte differentiation and proliferation. We make a summary of ALA-PDT inhibit Fibroblast Growth Factor-10 induced keratinocyte differentiation and proliferation through ROS in Fibroblast Growth Factor Receptor-2b pathway in Fig. 7.

Our results first confirmed that ALA-PDT induce ROS to suppress IL1α and further inhibit the differentiation and proliferation of epidermal keratinocytes through PKC downstream in FGFR2b pathway. Furthermore, we found that ALA-PDT has effect on keratinocytes differentiation and proliferation regulation which has a role for acne and keratin related dermatoses treatment. Although a few previous studies evaluated the comedolytic effects of ALA-PDT, the precise molecular mechanism from hyperkeratosis aspect has remained unknown. The findings in our study represent early evidence clarifying these cellular mechanisms, or at least a partial explanation for the treatment of hyperkeratosis related dermatoses *in vivo*.

## Methods

### Cell culture and treatment of cells

The immortalized human epidermal keratinocyte–like cell line HaCaT were cultured in DMEM supplemented with 10% fetal bovine serum, 100U/ml penicillin, and 100 mg/ml streptomycin (all from Invitrogen) and seeded in 75 cm^2^ culture flasks at a density of 1 × 10^5^ cells/ml. HaCaT were subcultured by trypsinization and used between the 2th and 6th passages.

HaCaT cells were seeded in four plates and cultured under standard conditions to >70% confluency. Cell proliferation and differentiation was induced by added 100 ng/ml FGF-10 (R&D System, Minneapolis, MN) for 24 hours before ALA was added. For the photodynamic therapy, it was performed as previously described^37^. All groups were washed with phosphate-buffered saline and the medium was changed once a day. For elimination in the presence of ROS, the glutathione (GSH) (Beyotime Biotechnology, China) was preincubated for 1 hour before the ALA was added. For stimulation and elimination in the presence of protein kinase C (PKC), 1 μmol/L PKC activator (Beyotime Biotechnology) and 200 nmol/L PKC inhibitor (Beyotime Biotechnology) were preincubated for 1 hour before the FGF-10 was added. All groups were studied at 3 h after the last treatment.

### Flow cytometric evaluation of apoptosis

Cells were treated in the same way as that for DAPI staining assay. The treated cells were harvested by mechanical scraping with a rubber and washed with PBS, and then double-stained by using an Annexin V-FITC apoptosis detection kit (Beyotime Institute of Biotechnology, Nantong, Jiangsu, China). Samples were incubated at room temperature for 15 min in the dark with Annexin V and PI, and quantitatively analyzed by a FACS vantage SE flow cytometer (Becton Dickinson, San Jose, CA, USA).

### Cell proliferation assay

Cell proliferation was assayed using a CCK-8 Kit (Beyotime Institute of Biotechnology, Nantong, Jiangsu, China). In brief, 8000 cells/well were transferred into 96-well plates after digestion with trypsin, and five parallel wells were used for each group. After attachment, the cells were subjected to the different treatments, and then cultured for 24 hours in a 5% CO_2_ incubator at 37 °C. Subsequently, 10 μ L of CCK-8 was added to each well, and the cells were cultured for another 2 hours. Cell density was determined by measuring the absorbance at 450 nm using a Varioskan Flash (Thermo Scientific, USA).

### ELISA for determination of interleukin (IL)1α

To investigate the expression of IL1α, ELISA was performed. The culture supernatant was collected and centrifuged at 15,000 × g for 5 min to remove the particulate matter, and directly used in fresh tubes. The concentrations of IL1α in the medium were determined using commercially available enzyme-linked immunosorbent assay kits (R&D systems China Co., Ltd., Shanghai, PRC) according to the manufacturer’s protocol.

### RNA extraction and quantitative real-time RT-PCR

Total RNA was extracted from HaCaT with TRIzol (Invitrogen, USA) according to the manufacturer’s recommendations. Real time-PCR was performed in ABI Step one plus Real time-PCR system (ABI) using SYBR Green I real time-PCR Master Mix (QPK201, TOYOBO, Japan). We analyzed relative expression of IL1α. The expression level of the studied genes was normalized to GAPDH as housekeeping control. The primer sequences for IL1α were 5′-GAAGAGACGGTTGAGTTTAAGCC-3′ (forward), and 5′-CAGGAAGCTAAAAGGTGCTGA-3′ (reverse). Data were analyzed using SDS Software 2.4 (Applied Biosystem).

### Measurement ofintracellular ROS

Intracellular production of ROS by HaCaT cells was measured by the oxidation of DCFH-DA to DCF. DCFH-DA is a non-polar compound that readily diffuses into cells, where it is hydrolyzed to the non-fluorescent polar derivative, DCFH, and thereby trapped within cells. When DCFH-DA is oxidized, it turns into the highly fluorescent DCF. Cells were incubated in the dark for 20 min at 37 °C with 10 mM DCFH-DA (Beyotime, Haimen, China) then harvested, and resuspended in PBS. Fluorescence was analyzed using a FACScan (Becton Dickinson, NJ, USA) flow cytometer with excitation at 488 nm and emission at 525 nm and the cells were examined under a confocal microscopy.

### Immunofluorescence and confocal microscopy for detection of keratin (K)1, K6, K16, FGFR2b, and PKC expressions in HaCaT

The cells were washed with 0.01M PBS and fixed in 4% formaldehyde for 30 min at room temperature. Cells were then incubated for 1 h with the following primary antibodies: rabbit polyclonal Bek (1:100 in PBS, Santa Cruz, CA), rabbit polyclonal Anti-Cytokeratin 1 (1:500, Abcam, Shanghai, China), rabbit monoclonal Anti-Cytokeratin 6 (1:100, Abcam), rabbit polyclonal Anti-Cytokeratin 16 (1:100, Abcam), and rabbit polyclonal Anti-PKC antibody (1:100, Abcam). The primary antibodies were visualized using goat anti-rabbit IgG-FITC (1:500 in PBS, Beyotime). Nuclei were stained with DAPI. Finally, the cover slips were mounted on the slides and fluorescence was visualized using a confocal laser fluorescence microscope (Carl Zesis Zen 2008, Carl Zeiss Inc., Germany). Photographic images were taken from five random fields.

### Western blotting assay

The cells were lysed in 62.5 mMTris–HCl (pH 6.8) containing 2% SDS, and the protein concentration was determined using the Pierce BCA assay (Thermo Fisher Scientific, Rockford, IL). Mercaptoethanol and bromophenol blue were added to make the final composition equivalent to the Laemmli sample buffer. The samples were fractionated using SDS-polyacrylamide gel electrophoresis (SDS-PAGE) and blotted onto Immobilon-P membrane (Millipore, Billerica, MA,). Goat anti-rabbit HRP (1:5000, Beyotime) was used as secondary antibodies. Antibody binding was visualized via Pierce ECL reagents (Thermo Fisher Scientific, CA). We used rabbit polyclonal Bek (1:500, Santa Cruz, CA), rabbit polyclonal Anti-Cytokeratin 1 (1:1000, Abcam), rabbit monoclonal Anti-Cytokeratin 6 (1:500, Abcam), rabbit polyclonal Anti-Cytokeratin 16 (1:500, Abcam), rabbit polyclonal Anti-PKC antibody (1:500, Abcam) as primary antibodies, and monoclonal GAPDH antibody (1:1000, Beyotime) as controls. The quantification of protein bands was established using Band-Scan software (PROZYME, San Leandro®, California).

### Animal models and ALA-PDT treatment

All animal experiments were conducted in accordance with the committee guidelines of the Nanjing Medical University and approved by the Animal Use Committee of Nanjing Medical University. Twenty New Zealand white female rabbits weighting 2.7–3.0 kg were obtained from the Experimental Animal Center of Jiangsu China. Rabbits were randomly divided to four groups (5 animals in each group): Control, ALA-PDT, OA, ALA-PDT + OA. The hyperkeratosis acne models were created using topical 50% oleic acid (OA) to ventral side of ear once daily for 14 days. After that, ALA-PDT was applied to the lesion using a piece of medical cotton approximately 2.0 cm^2^ in size to cover each ear. Each cotton piece was soaked with a specific concentration of ALA solution (20% (236 mg/mL); Fudan-Zhangjiang Bio-Pharmaceutical Co., Shanghai, China). Subsequently, the treated areas were covered with layers of plastic wrap and black plastic sheeting, secured with medical tape. Three hours later, these dressings were removed, and the treated tissue irradiated using a PDT laser (XD-635AB; Fudan-Zhangjiang Bio-Pharmaceutical) of wavelength 635 nm, duration 20 min, and energy density 60 J/cm^2^. This treatment was given once a week for a total of two treatments.

### Dermoscopy image

To evaluate the follicular hyperkeratosis lesions, images were taken by digital epiluminescence dermatoscope with ×200 magnification (Dinolite, Naarden, The Netherlands) at 7 days after the completion of treatment. Follicle was assessed using a physician global assessment score that grades the difference in the infra millimeter border.

### Histological and Immunohistochemical analysis

At 7 days after the completion of treatment, 4 mm punch skin biopsy specimens were taken from 20 rabbits. Skin biopsy specimens were fixed in 10% formalin, embedded in paraffin, and cut into 4 mm-thick sections. Sections were stained with hematoxylin-eosin or processed for immunohistochemical analysis. For the immunohistochemical staining, primary antibody rabbit polyclonal Bek (1:50), rabbit polyclonal Anti-PKC antibody (1:250), rabbit polyclonal Anti-Cytokeratin 1 (1:50, Abcam), rabbit monoclonal Anti-Cytokeratin 6 (1:20, Abcam), rabbit polyclonal Anti-Cytokeratin 16 (1:20, Abcam) were reacted for overnight at 4 °C, and secondary antibody goat anti-rabbit IgG-FITC (1:500 in PBS, Beyotime) was reacted for 1 hour at room temperature. Representative images were taken by confocal microscope.

### Statistical analysis

Statistical analysis was performed by using SPSS for Windows version 16.0 (SPSS, Chicago, IL, USA). The statistical analysis was carried out by One-way analysis of variance (ANOVA). Data are expressed as the mean ± SD for each group. Values of *P < 0.05, **P < 0.01, ***P < 0.001 were considered statistically significant and are indicated in the figures.

## Additional Information

**How to cite this article**: Gozali, M. V. *et al*. Photodynamic therapy inhibit Fibroblast Growth Factor-10 induced keratinocyte differentiation and proliferation through ROS in Fibroblast Growth Factor Receptor-2b pathway. *Sci. Rep.* **6**, 27402; doi: 10.1038/srep27402 (2016).

## Supplementary Material

Supplementary Information
