# Supplementary material for: Photodynamic therapy inhibit Fibroblast Growth Factor-10 induced keratinocyte differentiation and proliferation through ROS in Fibroblast Growth Factor Receptor-2b pathway
Source: Sci Rep. 2016 Jun 7;6:27402. doi: 10.1038/srep27402 (PMC4895211; doi:10.1038/srep27402)
Supplement: Supplementary Information [file srep27402-s1.pdf]

Photodynamic therapy inhibit Fibroblast Growth Factor-10 induced keratinocyte differentiation and proliferation through ROS in Fibroblast Growth Factor Receptor-2b pathway

Maya Valeska Gozali[1], Fei Yi[1], Jia-an Zhang[1], Juan Liu, Hong-jin Wu, Yang Xu, Dan Luo**, Bing-rong Zhou*

[1]These authors contributed equally to this work

Department of Dermatology, the First Affiliated Hospital of Nanjing Medical University, Nanjing, 210029, China

Supplementary figure

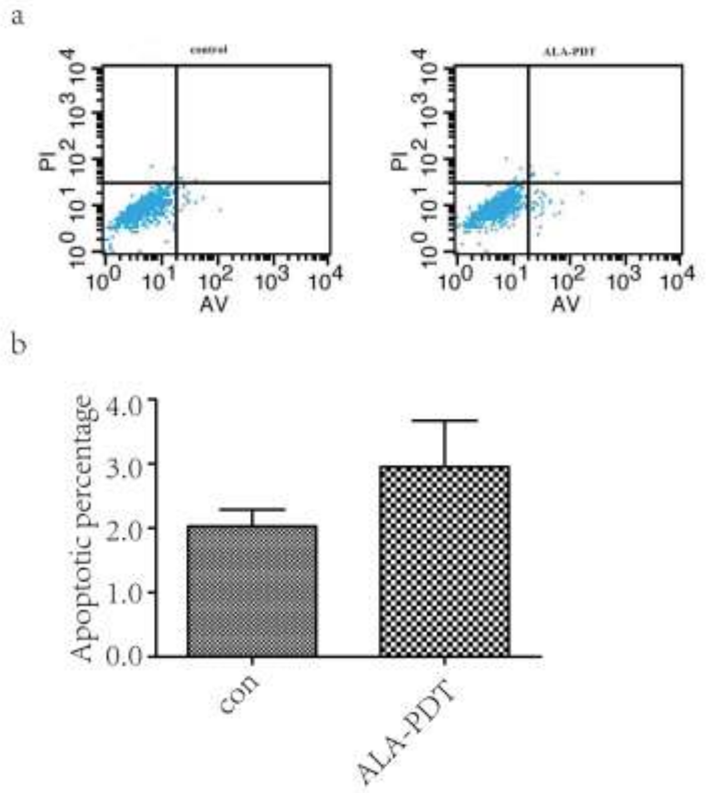

Supplementary figure legend. Evaluation of apoptotic in HaCaT cells

Flow cytometry were performed in 2 groups: control and ALA-PDT (1mM ALA and 3 J/cm^2) to observe whether ALA-PDT treatment has apoptotic effect to the HaCaT cells. Apoptotic percentage was quantified (a, b) and compared to control (2,29%), ALA-PDT only has a little apoptotic effect to the cells (3,67%). However, the differences were not significant ($P>0.05$).
